# Identification of Eastern United States *Reticulitermes* Termite Species via PCR-RFLP, Assessed Using Training and Test Data

**DOI:** 10.3390/insects6020524

**Published:** 2015-06-09

**Authors:** Ryan C. Garrick, Benjamin D. Collins, Rachel N. Yi, Rodney J. Dyer, Chaz Hyseni

**Affiliations:** 1 Department of Biology, University of Mississippi, Oxford, MS 38677, USA; E-Mails: bcollins@uams.edu (B.D.C.); ryi@umc.edu (R.N.Y.); chyseni@go.olemiss.edu (C.H.); 2 Department of Biology, Virginia Commonwealth University, Richmond, VA 23284, USA; E-Mail: rjdyer@vcu.edu

**Keywords:** dead wood, Isoptera, mitochondrial DNA, molecular diagnostics

## Abstract

*Reticulitermes* termites play key roles in dead wood decomposition and nutrient cycling in forests. They also damage man-made structures, resulting in considerable economic loss. In the eastern United States, five species (*R. flavipes*, *R. virginicus*, *R. nelsonae*, *R. hageni* and *R. malletei*) have overlapping ranges and are difficult to distinguish morphologically. Here we present a molecular tool for species identification. It is based on polymerase chain reaction (PCR) amplification of a section of the mitochondrial *cytochrome oxidase subunit II* gene, followed by a three-enzyme restriction fragment length polymorphism (RFLP) assay, with banding patterns resolved via agarose gel electrophoresis. The assay was designed using a large set of training data obtained from a public DNA sequence database, then evaluated using an independent test panel of *Reticulitermes* from the Southern Appalachian Mountains, for which species assignments were determined via phylogenetic comparison to reference sequences. After refining the interpretive framework, the PCR-RFLP assay was shown to provide accurate identification of four co-occurring species (the fifth species, *R. hageni*, was absent from the test panel, so accuracy cannot yet be extended to training data). The assay is cost- and time-efficient, and will help improve knowledge of *Reticulitermes* species distributions.

## 1. Introduction

Termites in the genus *Reticulitermes* provide critical ecosystem services in forests [1]. They also cause extensive damage to man-made structures, and are one of the most economically important pests in urban areas. Five species with overlapping geographic ranges occur in the eastern United States (*i.e*., *R. flavipes* Kollar, *R. virginicus* Banks, *R. nelsonae* Lim and Forschler, *R. hageni* Banks and Snyder, and *R. malletei* Howard and Clement), and they are notoriously difficult to distinguish morphologically [2]. In this group, the most informative characters are possessed by soldiers and adult winged reproductives (alates). However, soldiers are often quite rare (<5% frequency [3]), and even when they are encountered, 9–29 individuals are usually needed to achieve 95% confidence in species identification [2]. Furthermore, many colonies are quite young [4] such that only one primary reproductive pair (*i.e*., the original king and queen that founded the colony) is present, making this caste difficult to sample. Accordingly, molecular approaches offer considerable value for identification of *Reticulitermes* species, as they can be readily applied to the worker caste [5].

Mitochondrial DNA (mtDNA) sequences have been successfully used for species identification across diverse invertebrate groups (e.g., Collembola [6], Lepidoptera [7], and Hymenoptera [8]). Properties of this genome that simplify its utility include haploid uniparental inheritance, lack of recombination, and fast molecular evolutionary rates relative to protein-coding nuclear genes [9]. These advantages, coupled with the early development of conserved polymerase chain reaction (PCR) primers [10,11], have contributed to a strong representation of arthropod mtDNA sequences in public databases. *Reticulitermes* termites are no exception (e.g., currently >790 records for the five eastern United States species in the GenBank repository; www.ncbi.nlm.nih.gov). This large amount of information facilitates discovery of diagnostic nucleotide differences, which can then be targeted in genetic screening assays to enable identification of specimens regardless of caste or life stage.

Over the past two decades, natural resource managers have gained valuable information by using PCR-based molecular diagnostics for rapid taxonomic identification (e.g., illegal wildlife trade [12], biodiversity surveys [13], and trophic interactions [14]). Ultimately, these approaches are underpinned by DNA sequence-based comparison of an “unknown” sample to a reference database comprised of specimens of known identity. One potential limitation, however, relates to the cost of DNA sequencing. Indeed, when the pool candidate species to which a sample may belong is small and well-characterized, the information content of a typical Sanger sequence read (≥700-bp) can far exceed that needed to make a positive identification. In these cases, more cost- and time-efficient assays such as PCR-restriction fragment length polymorphism (RFLP) are desirable.

PCR-RFLP has been widely employed in studies focused on either intra- or interspecific levels of biological organization [15,16,17,18,19], including in the context of *Reticulitermes* species identification [3,20]. Here we extend on previous work by identifying a set of mtDNA sequence differences, located within restriction enzyme recognition sites, which can be used to distinguish among *Reticulitermes* species that co-occur in the eastern United States. We focused on the mitochondrial *cytochrome oxidase subunit II* (mt*COII*) gene because it is well represented in public databases, and compared to other genes such as the *cytochrome oxidase subunit I* (mt*COI*) “barcoding” gene region [13], it shows low intraspecific variability in *Reticulitermes* [2]. We applied the PCR-RFLP assay to an independent “test panel” (*i.e*., samples that had not contributed to primer design or selection of restriction enzymes) comprised of *Reticulitermes* collected from the Southern Appalachian Mountains. To assess accuracy, PCR-RFLP species identifications for the test panel were compared to identifications based on DNA sequencing coupled with phylogenetic analyses that included reference sequences for which morphological species diagnoses had been previously been performed. Finally, we refined the interpretive framework relating to expected PCR-RFLP banding patterns.

## 2. Materials and Methods

### 2.1. Database Mining and PCR-RFLP Assay Design

Mitochondrial *COII* sequences from each of the five target species of *Reticulitermes* were retrieved from the GenBank repository. Only those accessions that were reported to have been sampled from the eastern United States (broadly defined as east of the Mississippi River) were used. We also excluded sequences that contained premature stop codons, or were atypical of the focal species and thus may represent cases of nuclear-mitochondrial pseudogenes, or misidentification. Following this filtering step, for each focal species, mt*COII* sequences were aligned in MEGA v6.06 [21] and a consensus sequence containing information on all observed intraspecific nucleotide polymorphisms was generated. Species-specific consensus sequences were then aligned, and potentially diagnostic single nucleotide polymorphisms (SNPs) were identified.

NEBcutter v2.0 [22] was used to determine which of the potentially diagnostic DNA sequence differences could be resolved using restriction enzymes. The final choice of enzymes considered cost, simplicity of expected banding patterns (*i.e*., total number of cut sites and resolvability of fragment length differences via agarose gel electrophoresis), and overall efficiency (*i.e*., minimum number of separate restriction digest reactions). We then designed PCR primers (RetCo2-F: 5'-TGCAATACCATCCTTACG-3' and RetCo2-R: 5'-TCCACAGATTTCTGAGC-3') to amplify the most information-rich fragment of mt*COII* while keeping amplicon length short, so that electrophoretic separation of PCR-RFLP fragments was rapid. This also facilitates application to degraded DNA.

### 2.2. Empirical Application of the PCR-RFLP Assay to Novel Samples

**Figure 1:**
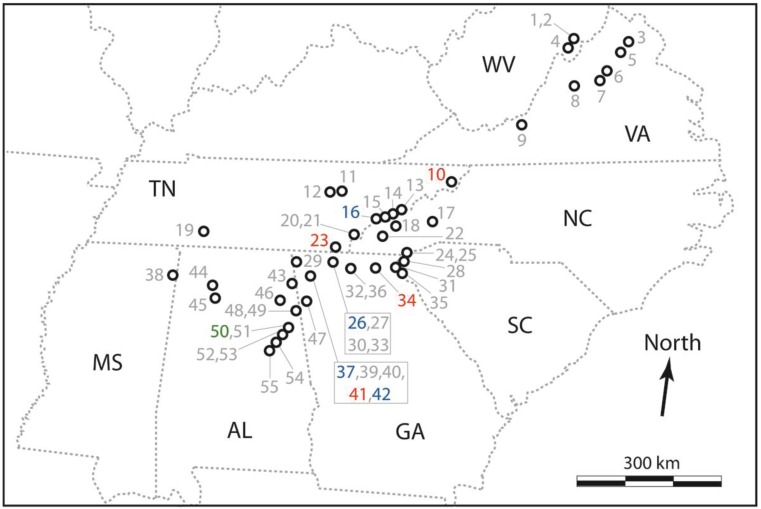
Map of collection sites of *Reticulitermes* specimens used as a “test panel” when assessing performance of the new PCR-RFLP assay. Sites are represented as black circles, numbered sequentially from 1–55 according to latitude, corresponding with Table S1 (grey numbers indicate sites from which *R. flavipes* were sampled, red = *R. virginicus*, blue = *R. malletei*, and green = *R. nelsonae*). States are represented by dashed grey lines, and those from which termites were collected are labelled with abbreviations (black text) as follows: Mississippi, MS; Alabama, AL; Georgia, GA; Tennessee, TN; South Carolina, SC; North Carolina, NC; Virginia, VA; and West Virginia, WV.

*Reticulitermes* termites were collected from 55 sites (*i.e*., decomposing logs) throughout the Southern Appalachian Mountains and surrounding forested areas Figure 1. Collection sites spanned Mississippi, Alabama, Georgia, Tennessee, South Carolina, North Carolina, Virginia and West Virginia because these states encompass geographic distributions of each of the five focal species including areas where they co-occur. We sampled natural populations (rather than infestations of man-made structures in urban areas) because these may contain a better representation of extant intraspecific genetic diversity. Following collection, specimens were stored in 95% ethanol, and spatial coordinates of each site were recorded (Table S1).

Genomic DNA was extracted from whole specimens (worker caste; one individual per site, *n* = 55) using a DNeasy Blood and Tissue Kit (Qiagen, Valencia CA, USA), following the manufacturer’s recommendations. The mt*COII* fragment was amplified in 10 µL volumes (or multiples thereof) comprised of 2.0 μL 5 × PCR buffer (Promega, Maddison WI, USA), 0.8 μL MgCl_2_ (25 mM, Promega), 1.6 μL dNTPs (1.25 mM, Promega), 0.5 μL Bovine Serum Albumin (10 mg/mL, New England BioLabs, Ipswich, MA, USA), 3.0 μL dH_2_O, 0.5 μL of each primer (RetCo2-F and RetCo2-R, 10 μM), 0.1 μL Go-*Taq* (5 U/μL, Promega), and 1.0 μL of genomic DNA. Amplifications were performed using a Bio-Rad T100 Thermal Cycler with the following profile: 95 °C for 2 min (1 cycle), 95 °C for 30 s, 54 °C for 30 s, and 72 °C for 1 min (35 cycles), and final extension of 72 °C for 2 min (1 cycle). Restriction enzyme digestions of PCR products were performed in 25 μL reaction volumes containing 13.75 μL dH_2_O, 2.5 μL of the recommended 10× restriction enzyme buffer (Promega), 0.25 μL Bovine Serum Albumin (10 mg/mL, New England BioLabs), 1.5 μL restriction enzyme (10 U/μL, Promega), and 7 μL of PCR product. Reactions were incubated at the recommended temperature overnight, to ensure complete digestion. For electrophoretic separation of fragments, we used 1.5% agarose gels with GelRed™ nucleic acid stain (Phenix Research Products, Asheville NC, USA), and 1× TBE buffer. A 100-bp ladder was included, and gels were run for ~3 h at 80 V, 50 mA.

### 2.3. Evaluation of Performance and Refinement of the Interpretive Framework

To assesses accuracy of the new PCR-RFLP assay, screened specimens were assigned to one of the five focal species via molecular phylogenetic comparison (described below) to Lim and Forschler’s [2] set of morphologically and genetically verified reference specimens of eastern *Reticulitermes* spp. (GenBank accessions: *R. flavipes*, EU689009; *R. virginicus*, EU689027; *R. hageni*, EU689026; *R. malletei* JF796227; and *R. nelsonae*, EU689013). First, a ~650-bp fragment of the mt*COII* gene was amplified using the same reaction mixture and thermal cycling conditions as described above, but using primers 5'-AGAGCWTCACCTATTATAGAAC-3' [23] and 5'-GTTTAAGAGACCAGTACTTG-3' [11], and a 50 °C annealing temperature. PCR products were purified using ExoSAP-IT^®^ (Affymetrix, Santa Clara, CA, USA), and sequenced on an Applied Biosystems 3730x Genetic Analyzer at Yale University. Next, chromatograms were edited and aligned in GENEIOUS v6.1.6 [24]. To confirm that data from true mtDNA had been obtained, rather than nuclear-mitochondrial pseudogenes which can be common in invertebrates [25], sequences were translated into amino acids and compared to accessions in NCBI’s nucleotide and protein databases via the BLAST search function [26].

Phylogenetic comparison of non-redundant mt*COII* sequences from the 55 new (this study) *vs.* five reference [2] *Reticulitermes* specimens was performed with MEGA using two different optimality criteria: maximum likelihood (ML) and maximum parsimony (MP). For the ML analysis, we used the best-fit model of nucleotide substitution identified using jModelTest 2 [27], empirical base frequencies, a Neighbor Joining starting tree, and nearest-neighbor-interchange branch swapping. MP analysis used 10 random addition starting trees, and tree-bisection-regrafting branch swapping. The pool of equally parsimonious trees was summarized as a 50% majority-rule consensus tree. For both optimality criteria, node support was assessed via 500 bootstrap replicates, and no out-group was included given that our main focus was on clustering of sequences, not on polarity of the branching process across the tree. Species identification of new specimens was based on their phylogenetic clustering with a reference sequence. As a working definition, the most inclusive and well-supported clade (bootstrap support >70% [28]) that contained only one reference sequence was considered to represent that particular *Reticulitermes* species.

For our test panel of 55 *Reticulitermes* termites, performance of the PCR-RFLP assay was evaluated in two ways. First, the proportion of unambiguous *vs.* ambiguous species assignments, given the expected banding patterns derived from GenBank accessions, was calculated. Second, we quantified the level of agreement between RFLP- *vs.* sequence-based species assignments. We then reconciled differences between RFLP- *vs.* DNA sequence-based species assignments by refining the interpretive framework to accommodate novel banding patterns seen during application of the PCR-RFLP assay.

## 3. Results

### 3.1. Database Mining and PCR-RFLP Assay Design

There were 132 GenBank accessions that met the conditions for inclusion in designing the new PCR-RFLP assay. However, representation of the five focal species was asymmetrical (*i.e*., 99 *R.* *flavipes*; 14 *R. virginicus*; 8 *R. nelsonae*; 7 *R. hageni*; and 4 *R. malletei*; Table S2). Based on the aligned consensus sequences (658-bp), 10 polymorphic sites that were potentially diagnostic of species were identified, and six could be resolved using commercially-available restriction enzymes (Table S3). We targeted four of these with three restriction enzymes: *Rsa* *I*, *Taq* *I*, and *Msp* *I*. The PCR primers that we designed (RetCo2-F and RetCo2-R) amplify a 376-bp mt*COII* fragment that contained these recognition sites. Expected banding patterns, based on *in silico* digestion of GenBank accessions, were relatively simple (*i.e*., maximum of three cut sites). Furthermore, fragments were of sufficiently different electrophoretic mobility that they could be resolved using standard agarose gels (*i.e*., minimum size differences of 12-, 10- and 37-bp for *Rsa I*, *Taq I* and *Msp I*, respectively, for fragments that are at least 50-bp long; Table S4).

### 3.2. Empirical Application of the PCR-RFLP Assay to Novel Samples

From the 55 *Reticulitermes* termites sampled from the Southern Appalachian Mountains (Figure 1; Table S1), the PCR-RFLP assay and interpretive framework designed from “training data” obtained from GenBank (Table S4) identified 37 *R. flavipes*, four *R. virginicus*, and four *R. malletei*. The remaining 10 specimens (18%) had an ambiguous species assignment. Ambiguities were attributable either to an unexpected three-enzyme combination of otherwise predicted banding patterns (*n* = 2 samples, each different from the other), or the unexpected absence of single-enzyme cut site (*n* = 8 samples, same missing cut site).

### 3.3. Evaluation of Performance and Refinement of the Interpretive Framework

**Figure 2:**
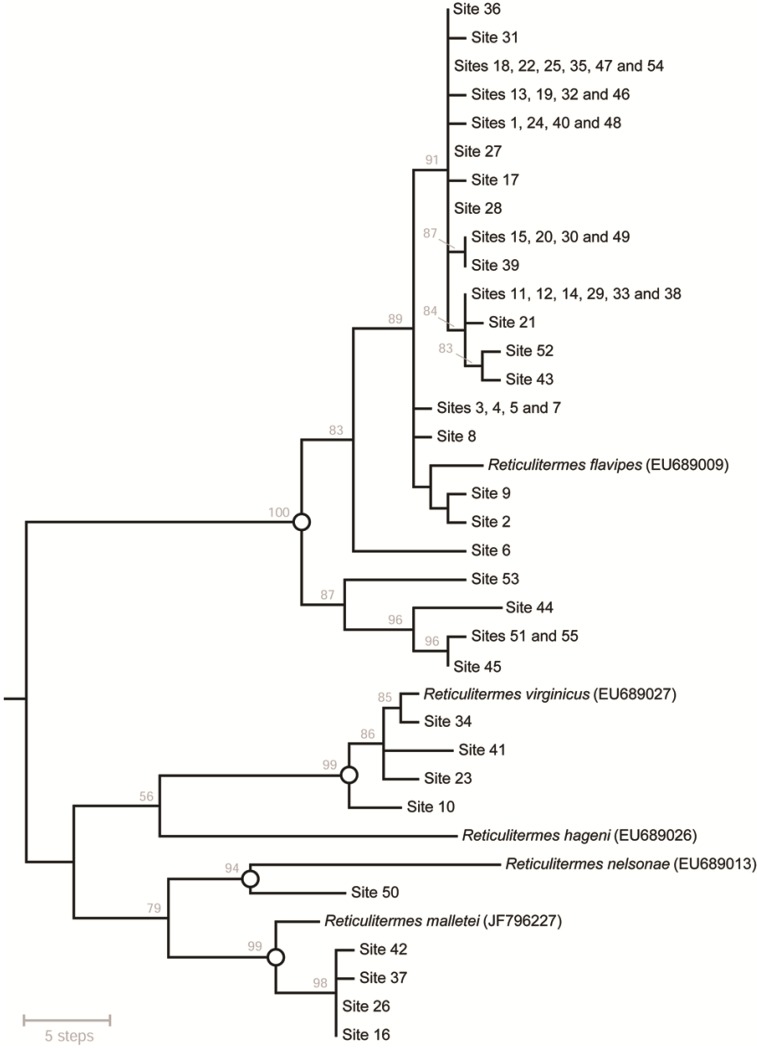
Phylogenetic relationships among *Reticulitermes* mt*COII* sequences from 55 new and five reference specimens (*n* = 37 non-redundant haplotypes; 607-bp alignment), estimated using Maximum Parsimony. Shown here is a 50% majority-rule consensus tree of eight equally-parsimonious trees (length = 166 steps). Tip labels indicate the geographic location(s) from which a termite with a given DNA sequence haplotype was sampled (Figure 2; Table S1). Numbers above nodes are bootstrap support values. Open circles on nodes mark the most inclusive well-support clades that contain only one reference taxon (the same groups were recovered using Maximum Likelihood; Figure S1).

For the all Southern Appalachian *Reticulitermes* termites assayed via PCR-RFLP, a 607-bp mt*COII* sequence alignment comprising 88 variable sites was generated (GenBank accessions: KR870353–KR870407). DNA sequences were AT-rich (63%), no premature stop codons were detected, the majority of substitutions (85.2%) were synonymous, and BLAST searches returned close matches to arthropod mt*COII*. Taken together, this indicated that true mtDNA had been sequenced. Thirty two non-redundant haplotypes were identified. Phylogenetic comparison with reference sequences from five eastern United States *Reticulitermes* termite species [2] was based on 106 variable sites (65 were parsimony-informative). For MP analysis, eight equally parsimonious trees (length = 166 steps) were obtained Figure 2. For ML analysis, the best-fit model of nucleotide substitution was HKY + I, and the best tree had a log likelihood score of −ln*L* = −1719.475 (Figure S1). Both optimality criteria produced the same outcomes with respect to species-level identification of Southern Appalachian specimens.

The majority (*n* = 46) of Southern Appalachian samples were phylogenetically assigned to *R. flavipes*. Remaining samples were comprised of *R. virginicus* and *R. malletei*, each represented by four specimens, plus one *R. nelsonae* sample (*R. hageni* was not present in our test panel). For *R. flavipes*, *R. virginicus* and *R. malletei*, species assignment was unambiguous; mean DNA sequence similarity between reference sequences *versus* the Southern Appalachian samples in their clade was 99%. Conversely, some uncertainty was associated with identification of *R. nelsonae*; the reference sequence showed lower (97%) mean DNA sequence similarity with the one Southern Appalachian sample assigned to this taxon. Thus, in this particular case we cautiously consider phylogenetic species identification to be tentative.

Comparison of PCR-RFLP *versus* phylogenetic species identification showed that the original interpretive framework (Table S4) had very low type I error, as no false positives were generated. However, for *R. flavipes*, type II error (defined here as uncertain species identification) was 19.6%. As noted above, this was due to either an unexpected three-enzyme combination of otherwise predicted banding patterns, or the unexpected absence of a single-enzyme cut site. If we tentatively accept that one *R. nelsonae* was present in our test panel of Southern Appalachian samples, then for that taxon, the assay would be considered to have a type II error of 100% given that this specimen had an ambiguous PCR-RFLP species assignment.

In light of the new mt*COII* sequence data contained within our test panel, we refined the interpretive framework associated PCR-RFLP banding patterns so as to accommodate previously unreported DNA sequence polymorphisms. These modifications entailed expanding the suite of expected banding patterns for one restriction enzyme for two taxa (*i.e*., *Taq I* for *R. flavipes*, and *Msp I* for *R. nelsonae*). As a result, all known type II error was eliminated. The refined interpretive framework is presented in Table 1.

**Table 1:** Refined interpretive framework for identifying eastern United States *Reticulitermes* species using PCR-RFLP, applied to the mt*COII* gene. Expected fragment sizes produced by three restriction digests (performed one-at-a-time) for each of five taxa are represented by check marks. While ≥2 banding patterns are possible for a given restriction enzyme in some taxa, all three-enzyme combinations are species-specific.

Restriction Enzyme	Fragment	Species				
	Sizes (bp)	*R. flavipes*	*R. hageni*	*R. malletei*	*R. nelsonae*	*R. virginicus*
*Rsa I*	175, 201	✓	〤	〤	✓	✓
	48, 127, 201	〤	〤	✓	✓	✓
	86, 115, 175	✓	✓	〤	〤	〤
*Taq I*	376	〤	✓	✓	✓	✓
	153, 223	〤	〤	〤	✓	〤
	183, 193	✓	〤	〤	〤	〤
	67, 126, 183	✓	〤	〤	〤	〤
	30, 67, 126, 153	✓	〤	〤	〤	〤
*Msp I*	376	✓	〤	〤	✓	✓
	37, 339 *	✓	〤	✓	〤	〤
	77, 299	〤	✓	〤	✓	〤
	37, 40, 299	〤	〤	✓	〤	〤

* For *R. flavipes*, fragment sizes may instead be 38-bp and 338-bp, but since the 1-bp differences compared to those reported in the table are indistinguishable, only the shorter fragments are listed.

## 4. Discussion

We developed a cost- and time-efficient three-enzyme PCR-RFLP assay that successfully identifies four of the five co-occurring species of eastern United States *Reticulitermes* (*R. hageni* was absent from the test panel, and thus accuracy could not be fully assessed; Table 1). This represents an advance on previous work, in part owing to: (1) our ability to leverage what is now a large body of publically available mtDNA sequence data, (2) our approach of deliberately separating two phases of the design process (training data *vs.* test data), and (3) the inclusion of recently described species (*R. nelsonae*).

Recent entomological applications of PCR-RFLP to resolving known mtDNA polymorphisms that aid in species identification have focused on the mt*COI* gene (e.g., Coleoptera [29], Hemiptera [30], Hymenoptera [31], and Lepidoptera [32]). For *Reticulitermes* termites, however, there is currently a far greater representation of mt*COII* sequences in public databases. Also, based on data from Cameron and Whiting’s [33] comparison of complete mitochondrial genomes from *R. flavipes* and *R. virginicus*, net sequence divergence (*i.e*., interspecific differences minus intraspecific differences) is greater for mt*COII* than for mt*COI* (mean of 6.15% *versus* 5.76%). Thus, the mt*COII* gene should be efficient for species identification in this group.

When PCR-RFLP is used for mtDNA-based species identification in insect groups, studies often identify species-specific SNPs (*i.e*., fixed nucleotide differences between taxa), which simplify interpretation of PCR-RFLP banding patterns. Notwithstanding the utility of mt*COII* for *Reticulitermes* species identification [3], intraspecific polymorphism was still quite high. For example, the *R. flavipes* consensus sequence (*i.e*., based on 99 accessions in the GenBank database; Table S3) contained 111 variables sites (17% of the 658-bp alignment). Previous studies have also reported that *Reticulitermes* mtDNA exhibits high polymorphism. For example, Luchetti *et al.* [34] suggested that the mt*COII* substitution rate may far exceed Brower’s [35] standard arthropod rate for cytochrome oxidase genes of ~2.3% sequence divergence between a pair of lineages per million years. Subsequently, Cameron and Whiting [33] extended findings of high substitution rates to the entire mtDNA genome of *Reticulitermes*. The main consequence for PCR-RFLP assay design is that, in this group, species-specific SNPs may be uncommon. However, this can be overcome by focusing instead on DNA sequence haplotypes (*i.e*., a series of physically linked SNPs).

The present study benefited from a large public database of *Reticulitermes* mt*COII* sequences. Nonetheless, additional intraspecific variation was discovered in Southern Appalachian termite samples. Based on the initial interpretive framework (Table S4), the proportion of ambiguous species assignments serves as a measure of the extent to which polymorphism in the 376-bp mt*COII* fragment, as it exists in natural populations, was not represented in the public DNA sequence database. Somewhat surprisingly, this type II error was quite pronounced for *R. flavipes*, despite the species being represented by 99 GenBank accessions spanning a geographic area encompassing >10 states. An earlier study by Szalanski *et al.* [3] focused on molecular diagnostics of *Reticulitermes* species using PCR-RFLP and included some of the same species in our set (*i.e*., *R. flavipes*, *R. hageni*, and *R. virginicus*), but was designed on a much smaller subset of sequences (*n* = 15 *vs.* *n* = 120 in the present study, considering only the shared species). Accordingly, advances provided by the PCR-RFLP assay reported here is warrants consideration.

The Szalanski *et al.* [3] PCR-RFLP assay was based on two mtDNA genes (*COII* and 16SrRNA) amplified individually, each digested with two different restriction enzymes. For comparative purposes, we focused on mt*COII* and performed an *in* *silico* application of their assay to our consensus sequences for *R. flavipes*, *R. hageni*, and *R. virginicus* (Table S3, but with the alignment trimmed to 379-bp, and digested with *Hinf I* and *Taq I*). We found that identification of *R. flavipes* may be more problematic than the authors originally anticipated. For example, owing to previously unappreciated polymorphism at two *Hinf I* recognition sites (their positions 97 and 184), *R. flavipes* would generate the same mt*COII* banding patterns as *R. hageni* and *R. virginicus*. Similarly, *R. flavipes* would be indistinguishable from the other two species due to polymorphism at a *Taq I* recognition site (their position 292). However, because Szalanski *et al.*’s [3] assay included a second gene, their assay would mostly likely lead to an ambiguous species assignment (*cf*. misidentification). Similar issues are likely to affect many PCR-RFLP assays, given the common underlying assumption that all extant polymorphism has been fully characterized. As this will almost never be true, we recommend sequencing of a subset of specimens coupled with phylogenetic comparison to reference specimens, even if PCR-RFLP species identification appears unambiguous. From an applied perspective, this could be focused on specimens from areas where previous sampling has been sparse (*i.e*., outside of the Southern Appalachians, and/or those that are poorly represented in GenBank), or wherever two or more *Reticulitermes* species are suspected to be sympatric.

The PCR-RFLP assay presented here provides a useful tool for studies of *Reticulitermes* species in the eastern United States. For example, phylogeographic investigations of spatial patterns of genetic diversity rely on dense sampling of individuals over a relatively broad geographic area [36,37], but a fundamental prerequisite is that only conspecifics are included in downstream analyses. In this respect, *Reticulitermes* termites have been challenging study organisms. However, once species identifications can be readily achieved, dead-wood associated forest invertebrates may be particularly good models for understanding organismal responses to past landscape-level environmental change [16,38,39,40], and for identifying montane biodiversity habitat refuges [41]. Previous mtDNA-based surveys of *R. flavipes* from parts of the eastern United States have revealed interesting phylogeographic patterns that demand further investigation [42,43]. Our PCR-RFLP assay should facilitate extensions of that work.

Wood-feeding insects such as *Reticulitermes* termites make important contributions to decomposition and nutrient cycling processes in forests [1], yet these ecosystem service providers have poorly known species distributions [35]. The new PCR-RFLP assay will help to redress this. For example, our sampling in the Southern Appalachian Mountains and surrounding areas appears to have extended the known range of *R. nelsonae*. Previously, this species was known only from Georgia, North Carolina, and Florida [2]. Assuming that our phylogenetic identification of this species is accurate (Figure 2; Figure S1), it appears that *R. nelsonae* also occurs in Alabama (Talladega National Forest, site 50; Figure 1; Table S1). When additional occurrence data have been accumulated, that information could be coupled with remote sensing or other environmental data to generate species distribution models [44] or to quantify degree of niche overlap [45]. Thus, the new PCR-RFLP assay could also help determine whether the five *Reticulitermes* species are ecologically differentiated.

## 5. Conclusions

*Reticulitermes* termites are functionally important ecosystem engineers. The new molecular diagnostic tool for identification of eastern United States *Reticulitermes* species facilitates new lines of investigation by providing a means by which to rapidly classify “unknown” samples prior to downstream analyses. The PCR-RFLP assay can be implemented without specialist equipment or expertise, and is cost-efficient. As public DNA sequence databases continue to be populated with new data from *Reticulitermes* species, periodic re-evaluation and refinement may be necessary. For now, however, the PCR-RFLP assay reported here should be broadly useful.

## Supplementary Files

Supplementary File 1

## References

[1] Ulyshen M.D. (2014). Interacting effects of insects and flooding on wood decomposition. PLoS ONE.

[2] Lim S.Y., Forschler B.T. (2012). *Reticulitermes nelsonae*, a new species of subterranean termite (Rhinotermitidae) from the southeastern United States. Insects.

[3] Szalanski A.L., Austin J.W., Owens C.B. (2003). Identification of *Reticulitermes* spp. (Isoptera: Reticulitermatidae) from south central United States by PCR-RFLP. J. Econ. Entomol..

[4] Vargo E.L. (2003). Hierarchical analysis of colony and population genetic structure of the eastern subterranean termite, *Reticulitermes flavipes*, using two classes of molecular markers. Evolution.

[5] Vargo E.L., Husseneder C. (2009). Biology of subterranean termites: Insights from molecular studies of *Reticulitermes* and *Coptotermes*. Annu. Rev. Entomol..

[6] Hogg I.D., Hebert P.D.N. (2004). Biological identification of springtails (Hexapoda: Collembola) from the Canadian Arctic, using mitochondrial DNA barcodes. Can. J. Zool..

[7] Hajibabaei M., Janzen D.H., Burns J.M., Hallwachs W., Hebert P.D.N. (2006). DNA barcodes distinguish species of tropical Lepidoptera. Proc. Natl. Acad. Sci. USA.

[8] Sheffield C.S., Hebert P.D., Kevan P.G., Packer L. (2009). DNA barcoding a regional bee (Hymenoptera: Apoidea) fauna and its potential for ecological studies. Mol. Ecol. Resour..

[9] Avise J.C., Arnold J., Ball R.M., Bermingham E., Lamb T., Neigel J.E., Reeb C.A., Saunders N.C. (1987). Intraspecific phylogeography: The mitochondrial DNA bridge between population genetics and systematics. Annu. Rev. Ecol. Syst..

[10] Folmer O., Black M., Hoeh W., Lutz R., Vrijenhoek R. (1994). DNA primers for amplification of mitochondrial cytochrome *c* oxidase subunit I from diverse metazoan invertebrates. Mol. Mar. Biol. Biotechnol..

[11] Simon C., Frati F., Beckenbach A., Crespi B., Liu H., Flook P. (1994). Evolution, weighting, and phylogenetic utility of mitochondrial gene sequences and a compilation of conserved polymerase chain reaction primers. Ann. Entomol. Soc. Am..

[12] Baker C.S., Cipriano F., Palumbi S.R. (1996). Molecular genetic identification of whale and dolphin products from commercial markets in Korea and Japan. Mol. Ecol..

[13] Hebert P.D.N., Cywinska A., Ball S.L., deWaard J.R. (2003). Biological identifications through DNA barcodes. Proc. R. Soc. Lond. B Biol. Sci..

[14] Jarman S.N., Deagle B.E., Gales N.J. (2004). Group-specific polymerase chain reaction for DNA-based analysis of species diversity and identity in dietary samples. Mol. Ecol..

[15] Garrick R.C., Meadows C.A., Nicolas A.N., Nason J.D., Dyer R.J. (2008). A set of polymorphic nuclear intron markers for conservation genetics and phylogeography of *Euphorbia* species (*Pedilanthus* clade). Conserv. Genet..

[16] Garrick R.C., Rowell D.M., Simmons C.S., Hillis D.M., Sunnucks P. (2008). Fine-scale phylogeographic congruence despite demographic incongruence in two low-mobility saproxylic springtails. Evolution.

[17] Garrick R.C., Meadows C.A., Nason J.D., Cognato A.I., Dyer R.J. (2009). Variable nuclear markers for a Sonoran Desert bark beetle, *Araptus attenuatus* Wood (Curculionidae: Scolytinae), with applications to related genera. Conserv. Genet..

[18] Garrick R.C., Nason J.D., Fernández-Manjarrés J.F., Dyer R.J. (2013). Ecological coassociations influence species’ responses to past climatic change: An example from a Sonoran Desert bark beetle. Mol. Ecol..

[19] Lancaster M.L., Gemmell N.J., Negro S., Goldsworthy S., Sunnucks P. (2006). Ménage à trois on Macquarie Island: Hybridization among three species of fur seal (*Arctocephalus* spp.) following historical population extinction. Mol. Ecol..

[20] Wang C., Zhou X., Li S., Schwinghammer M., Scharf M.E., Buczkowski G., Bennett G.W. (2009). Survey and identification of termites (Isoptera: Rhinotermitidae) in Indiana. Ann. Entomol. Soc. Am..

[21] Tamura K., Stecher G., Peterson D., Filipski A., Kumar S. (2013). MEGA6: Molecular Evolutionary Genetics Analysis version 6.0. Mol. Biol. Evol..

[22] Vincze T., Posfai J., Roberts R.J. (2003). NEBcutter: A program to cleave DNA with restriction enzymes. Nucleic Acids Res..

[23] Park Y.C., Maekawa K., Matsumoto T., Santoni R., Choe J.C. (2004). Molecular phylogeny and biogeography of the Korean woodroaches *Cryptocercus* spp.. Mol. Phylogenetics Evol..

[24] Kearse M., Moir R., Wilson A., Stones-Havas S., Cheung M., Sturrock S., Buxton S., Cooper A., Markowitz S., Duran C. (2012). Geneious Basic: An integrated and extendable desktop software platform for the organization and analysis of sequence data. Bioinformatics.

[25] Sunnucks P., Hales D.F. (1996). Numerous transposed sequences of mitochondrial cytochrome oxidase I–II in aphids of the genus *Sitobion* (Hemiptera: Aphididae). Mol. Biol. Evol..

[26] Altschul S.F., Gish W., Miller W., Myers E.W., Lipman D.J. (1990). Basic local alignment search tool. J. Mol. Biol..

[27] Darriba D., Taboada G.L., Doallo R., Posada D. (2012). jModelTest 2: More models, new heuristics and parallel computing. Nat. Methods.

[28] Hillis D.M., Bull J.J. (1993). An empirical test of bootstrapping as a method for assessing confidence in phylogenetic analysis. Syst. Biol..

[29] Germain J.-F., Chatot C., Meusnier I., Artige E., Rasplus J.-Y., Cruaud A. (2013). Molecular identification of *Epitrix* potato flea beetles (Coleoptera: Chrysomelidae) in Europe and North America. Bull. Entomol. Res..

[30] Ovalle T.M., Parsa S., Hernández M.P., Becerra Lopez-Lavalle L.A. (2014). Reliable molecular identification of nine tropical whitefly species. Ecol. Evol..

[31] Vesterlund S.-R., Sorvari J., Vasemägi A. (2014). Molecular identification of cryptic bumblebee species from degraded samples using PCR-RFLP approach. Mol. Ecol. Resour..

[32] Arimoto M., Iwaizumi R. (2014). Identification of Japanese *Lymantria* species (Lepidoptera: Lymantriidae) based on PCR-RFLP analysis of mitochondrial DNA. Appl. Entomol. Zool..

[33] Cameron S.L., Whiting M.F. (2007). Mitochondrial genomic comparisons of the subterranean termites from the Genus *Reticulitermes* (Insecta: Isoptera: Rhinotermitidae). Genome.

[34] Luchetti A., Mantovani B., Marini M. (2005). Mitochondrial evolutionary rate and speciation in termites: Data on European *Reticulitermes* taxa (Isoptera: Rhinotermitidae). Insectes Soc..

[35] Brower A.V.Z. (1994). Rapid morphological radiation and convergence among races of the butterfly *Heliconius erato* inferred from patterns of mitochondrial DNA evolution. Proc. Natl. Acad. Sci. USA.

[36] Garrick R.C., Caccone A., Sunnucks P. (2010). Inference of population history by coupling exploratory and model-driven phylogeographic analyses. Int. J. Mol. Sci..

[37] Garrick R.C., Bonatelli I.A., Hyseni C., Morales A., Pelletier T.A., Perez M.F., Rice E., Satler J.D., Symula R.E., Thomé M.T. (2015). The evolution of phylogeographic data sets. Mol. Ecol..

[38] Sunnucks P., Blacket M.J., Taylor J.M., Sands C.J., Ciavaglia S.A., Garrick R.C., Tait N.N., Rowell D.M., Pavlova A. (2006). A tale of two flatties: Different responses of two terrestrial flatworms to past environmental climatic fluctuations at Tallaganda in montane southeastern Australia. Mol. Ecol..

[39] Garrick R.C., Rowell D.M., Sunnucks P. (2012). Phylogeography of saproxylic and forest floor invertebrates from Tallaganda, south-eastern Australia. Insects.

[40] Bull J.K., Sands C.J., Garrick R.C., Gardner M.G., Tait N.N., Briscoe D.A., Rowell D.M., Sunnucks P. (2013). Environmental complexity and biodiversity: The multi-layered evolutionary history of a log-dwelling velvet worm in montane temperate Australia. PLoS ONE.

[41] Garrick R.C. (2011). Montane refuges and topographic complexity generate and maintain invertebrate biodiversity: Recurring themes across space and time. J. Insect Conserv..

[42] Szalanski A.L., Austin J.W., McKern J.A., Scheffrahn R.H., Owens C.B., Messenger M.T. (2008). Molecular phylogeography of *Reticulitermes* (Isoptera: Rhinotermitidae) termites from Florida. Sociobiology.

[43] Perdereau E., Bagnères A.-G., Bankhead-Dronnet S., Dupont S., Zimmermann M., Vargo E.L., Dedeine F. (2013). Global genetic analysis reveals the putative native source of the invasive termite, *Reticulitermes flavipes*, in France. Mol. Ecol..

[44] Elith J., Leathwick J.R. (2009). Species distribution models: Ecological explanation and prediction across space and time. Annu. Rev. Ecol. Evol. Syst..

[45] Edwards D.L., Garrick R.C., Tapia W., Caccone A. (2014). Cryptic structure and niche divergence within threatened Galápagos giant tortoises from southern Isabela Island. Conserv. Genet..

