# Supplementary material for: Identification of Eastern United States Reticulitermes Termite Species via PCR-RFLP, Assessed Using Training and Test Data
Source: Insects. 2015 Jun 9;6(2):524–37. doi: 10.3390/insects6020524 (PMC4553497; doi:10.3390/insects6020524)
Supplement: Supplementary File 1 [file insects-06-00524-s001.pdf]

Supplemental Information

Table S1. List of collection sites of *Reticulitermes* specimens used as a “test panel” when assessing performance of the new PCR-RFLP assay. Spatial coordinates are in decimal degrees, and elevation is reported in meters above sea level. State abbreviations are: Mississippi, MS; Alabama, AL; Georgia, GA; Tennessee, TN; South Carolina, SC; North Carolina, NC; Virginia, VA; and West Virginia, WV. Region abbreviations are: National Forest, NF; National Park, NP; State Forest, SF; State Park, SP; and Wildlife Management Area, WMA.

Site No.	Latitude	Longitude	Elevation	State	Region
1	38.82585	−79.38506	548	WV	Monongahela NF
2	38.82374	−79.38618	528	WV	Monongahela NF
3	38.80508	−78.18149	755	VA	Shenandoah NP
4	38.72694	−79.48494	936	WV	Monongahela NF
5	38.62592	−78.34060	1032	VA	Shenandoah NP
6	38.29123	−78.64308	761	VA	Shenandoah NP
7	38.12902	−78.78368	814	VA	Shenandoah NP
8	38.04052	−79.34980	784	VA	Washington NF
9	37.34757	−80.54509	1121	VA	Jefferson NF
10	36.31100	−82.07211	648	TN	Cherokee NF
11	36.13606	−84.48829	496	TN	Frozen Head SP
12	36.12452	−84.74478	378	TN	Clear Crk
13	35.77140	−83.21343	575	TN	Great Smoky Mtn NP
14	35.70232	−83.35717	653	TN	Great Smoky Mtn NP
15	35.65682	−83.51849	780	TN	Great Smoky Mtn NP
16	35.61933	−83.66993	593	TN	Great Smoky Mtn NP
17	35.59535	−82.48742	722	NC	Blue Ridge Pkwy
18	35.52117	−83.31077	666	TN	Great Smoky Mtn NP
19	35.39384	−87.52677	304	TN	Natchez Trace Pkwy
20	35.34883	−84.24760	327	TN	Cherokee NF
21	35.34534	−84.19383	425	TN	Cherokee NF
22	35.32969	−83.59187	593	NC	Nantahala NF
23	35.10896	−84.62477	530	TN	Cherokee NF
24	35.01376	−83.05563	887	SC	Sumter NF
25	34.94523	−83.08929	744	SC	Sumter NF
26	34.93135	−84.65486	485	GA	Chattahoochee NF
27	34.87866	−84.71137	354	GA	Chattahoochee NF
28	34.86200	−83.10755	536	SC	Oconee SP
29	34.84695	−85.49971	315	GA	Cloudland Canyon SP
30	34.77972	−84.63805	764	GA	Chattahoochee NF
31	34.77755	−83.31242	469	SC	Sumter NF
32	34.77507	−84.33880	730	GA	Chattahoochee NF
33	34.75931	−84.69140	804	GA	Chattahoochee NF
34	34.74192	−83.73265	766	GA	Chattahoochee NF
35	34.72782	−83.22783	394	SC	Sumter NF
36	34.68311	−84.25093	810	GA	Chattahoochee NF
37	34.64336	−85.21630	386	GA	Johns Mtn WMA

Table S1. *Cont.*

Site No.	Latitude	Longitude	Elevation	State	Region
38	34.60502	−88.19299	177	MS	Tishomingo SP
39	34.57297	−85.06536	450	GA	Johns Mtn WMA
40	34.56515	−85.24268	408	GA	Johns Mtn WMA
41	34.56416	−85.24043	427	GA	Johns Mtn WMA
42	34.54107	−85.25067	341	GA	Johns Mtn WMA
43	34.45540	−85.58357	395	AL	Little River Canyon
44	34.41979	−87.33273	321	AL	Bankhead NF
45	34.17659	−87.27680	248	AL	Bankhead NF
46	34.14676	−85.84679	188	AL	Shinbone Ridge
47	34.12260	−85.26428	232	GA	Lyons Bridge
48	33.96340	−85.45730	300	AL	Talladega NF
49	33.91858	−85.49764	257	AL	Talladega NF
50	33.57157	−85.69391	328	AL	Talladega NF
51	33.56059	−85.70074	425	AL	Talladega NF
52	33.47105	−85.80658	621	AL	Talladega NF
53	33.46215	−85.81731	485	AL	Talladega NF
54	33.40451	−85.87318	460	AL	Talladega NF
55	33.20150	−86.07201	291	AL	Talladega NF

Table S2. List of GenBank accessions of mt*COII* used for initial design of a PCR-RFLP assay for identification of eastern United States *Reticulitermes* termite species.

Species	State	GenBank accession(s)	Original reference
R. flavipes	DE	JQ280721–JQ280727	[1]
	FL	AF525321	[2]
	FL	AY808077, AY808082–AY808086	[3]
	FL	JQ280662–JQ280694, JQ280745–JQ280746	[1]
	GA	AF107479–AF107482, AF107484	[4]
	GA	AY027477	[5]
	GA	EF206316–EF206317	[6]
	GA	JQ280701	[1]
	IN	AY168203, AY168210–AY168211	[7]
	IN	JQ280705	[1]
	MA	DQ493725	[8]
	MS	JQ280622–JQ280626, JQ280741–JQ280744	[1]
	NC	DQ493728	[8]
	NC	JQ280716–JQ280720	[1]
	OH	JQ280702	[1]
	SC	JQ280711–JQ280715	[1]
	TN	JQ280703	[1]
	VA	JQ280728–JQ280736	[1]
	GA	JF796216	[9]

Table S2. *Cont.*

Species	State	GenBank accession(s)	Original reference
R. flavipes	GA	JF796217	[9]
	?	JF796218	[9]
	FL	JF796220	[9]
	GA	JN207492	[9]
R. hageni	GA	AF107486	[4]
	GA	AY027478	[5]
	GA	AF525328	[2]
	GA	EU689026	[10]
	NC	DQ493729	[8]
	GA	JF796224	[9]
	GA	JF796225	[9]
R. malletei	GA	GU550074	[10]
	GA	JF796226	[9]
	GA	JF796227	[9]
	NC	JF796228	[9]
R. nelsonae	GA	EU689013	[9]
	NC	JF796229	[9]
	GA	JF796230	[9]
	FL	JF796231	[9]
	GA	JF796232	[9]
	FL	JF796233	[9]
	GA	JF796235	[9]
	GA	JF796236	[9]
R. virginicus	FL	AF525356	[2]
	FL	AY808096	[3]
	FL	AY808098	[3]
	FL	EF206319	[6]
	GA	AF107487	[4]
	GA	AY027479	[5]
	GA	EF206318	[6]
	GA	EU689027	[10]
	NC	DQ493743	[8]
	VA	AF525357	[2]
	GA	JF796221	[9]
	GA	JF796222	[9]
	GA	JF796223	[9]
	FL	JF796234	[9]

Table S3. Consensus mt*COII* sequences for each five *Reticulitermes* termite species, showing polymorphic nucleotide positions within a 658-bp alignment. IUPAC ambiguity codes reflect intraspecific variability, based on the GenBank accessions from which consensus sequences were generated (Table S2). Polymorphisms that could potentially be used for species identification are color-coded according to whether only two alternative nucleotide states exist (red = one state is unique to a particular species, or green = one state is characteristic of a subset of species) *vs*. three alternative nucleotide states (blue). In each case, underlined nucleotides indicate potentially diagnostic character(s). The ✓ and ✗ symbols indicate whether DNA sequence polymorphisms do or do not generate restriction site polymorphisms, respectively. Dark grey shading indicates locations of PCR primers that we designed (*i.e.*, RetCo2-F and RetCo2-R) to amplify the most information-rich region (pale grey).

**Position (bp)**	**6**	**12**	**18**	**19**	**22**	**23**	**24**	**27**	**28**	**32**	**36**	**39**	**42**	**58**	**60**	**72**	**75**	**78**	**82**	**90**	**93**	**94**	**105**	**107**	**111**	**114**	**117**	**123**	**129**
R. flavipes	R	A	H	M	A	S	R	W	C	R	A	A	Y	Y	R	Y	Y	T	H	Y	R	T	Y	Y	C	T	A	R	Y
R. hageni	A	A	C	A	A	G	A	A	C	A	A	A	T	C	G	Y	C	Y	T	C	A	Y	C	C	C	A	R	A	T
R. malletei	A	R	C	A	A	G	A	A	C	A	R	G	T	C	G	T	C	T	T	Y	A	C	C	C	C	R	R	A	C
R. nelsonae	A	A	Y	A	A	G	A	A	C	A	W	A	Y	C	R	C	C	Y	T	T	A	C	C	C	C	A	R	A	Y
R. virginicus	A	A	C	A	R	G	A	W	S	A	A	A	C	C	R	Y	C	T	T	Y	A	T	Y	C	Y	A	G	A	C
Cut site												✗														✗			

	**135**	**137**	**138**	**141**	**144**	**147**	**150**	**152**	**153**	**162**	**163**	**165**	**166**	**171**	**177**	**178**	**186**	**189**	**195**	**201**	**202**	**207**	**213**	**217**	**219**	**228**	**230**	**231**	**234**
R. flavipes	R	Y	T	R	T	A	Y	Y	Y	Y	R	C	C	R	A	H	A	Y	R	T	R	A	Y	Y	R	T	C	A	A
R. hageni	A	T	Y	R	T	A	C	C	T	T	A	T	T	A	R	C	R	Y	A	T	G	A	C	C	A	T	C	A	A
R. malletei	A	T	T	A	T	A	C	C	C	C	A	T	T	A	A	M	A	C	A	T	G	A	C	C	A	T	C	G	A
R. nelsonae	A	T	T	A	Y	R	Y	C	T	Y	A	Y	Y	A	R	Y	R	C	A	Y	G	A	C	C	A	Y	C	R	R
R. virginicus	A	T	T	A	T	R	C	C	T	T	A	T	T	A	A	C	A	Y	A	T	G	R	C	C	A	T	S	R	A
Cut site																													

	**240**	**249**	**250**	**252**	**255**	**256**	**258**	**264**	**268**	**270**	**273**	**279**	**280**	**288**	**289**	**291**	**294**	**297**	**300**	**303**	**306**	**309**	**312**	**318**	**323**	**324**	**327**	**330**	**333**
R. flavipes	Y	Y	C	R	Y	Y	A	Y	A	Y	Y	A	A	Y	C	R	R	D	D	R	Y	R	A	A	D	T	A	Y	M
R. hageni	C	C	C	A	Y	C	A	C	A	C	C	R	A	T	C	A	A	A	A	A	C	A	G	R	A	T	A	Y	A
R. malletei	C	T	C	A	T	C	A	C	A	C	C	A	A	C	C	A	A	A	A	A	C	A	A	G	A	T	A	C	A
R. nelsonae	C	T	Y	A	T	C	A	C	W	Y	Y	A	R	C	Y	A	A	A	A	A	C	R	A	A	A	T	R	T	A
R. virginicus	Y	T	C	A	T	C	W	C	A	C	Y	A	A	C	C	A	A	A	R	A	C	A	A	R	A	Y	A	C	A
Cut site																							✓						

Tale S3. *Cont.*

**Position (bp)**	**339**	**348**	**354**	**360**	**363**	**369**	**372**	**375**	**381**	**382**	**388**	**389**	**394**	**396**	**399**	**406**	**408**	**417**	**420**	**421**	**426**	**430**	**432**	**441**	**444**	**450**	**454**	**456**	**458**
R. flavipes	Y	A	Y	Y	Y	C	R	R	W	W	S	C	R	Y	Y	Y	A	Y	T	C	T	H	A	Y	H	Y	C	R	Y
R. hageni	C	A	T	T	C	T	A	R	A	A	A	C	A	C	C	Y	A	C	C	C	Y	C	A	C	A	C	Y	A	T
R. malletei	C	A	T	T	C	T	A	A	A	A	G	C	A	C	C	T	A	C	C	C	T	C	A	T	A	C	T	A	T
R. nelsonae	C	R	T	T	C	T	R	A	A	A	R	C	R	C	C	T	R	Y	C	C	T	C	A	Y	A	Y	Y	A	T
R. virginicus	C	A	T	T	C	T	A	R	A	A	A	Y	A	C	C	C	A	C	C	S	T	C	S	T	A	C	T	A	T
Cut site						✓													✓				×						
	**462**	**474**	**477**	**478**	**480**	**483**	**484**	**486**	**495**	**504**	**507**	**510**	**513**	**519**	**522**	**525**	**528**	**531**	**534**	**535**	**540**	**544**	**545**	**546**	**551**	**552**	**554**	**556**	**561**
R. flavipes	R	Y	R	T	R	C	T	D	Y	D	D	R	R	Y	Y	R	A	R	A	C	Y	G	Y	A	Y	C	Y	W	Y
R. hageni	A	C	A	T	A	C	T	A	T	R	G	G	A	C	C	A	G	R	R	Y	T	W	C	A	T	C	C	A	T
R. malletei	A	C	A	C	A	C	T	A	T	A	R	G	A	C	C	A	R	A	A	T	T	G	C	A	T	C	C	A	T
R. nelsonae	A	C	R	C	A	C	Y	W	Y	R	R	G	A	C	Y	A	G	R	A	Y	T	A	C	A	T	Y	C	A	T
R. virginicus	A	C	A	T	A	Y	T	A	T	A	R	G	R	C	C	A	A	A	A	Y	T	A	M	M	T	C	C	A	T
Cut site				✓																									
	**564**	**567**	**571**	**573**	**574**	**578**	**579**	**585**	**597**	**600**	**604**	**606**	**609**	**612**	**618**	**620**	**621**	**622**	**624**	**627**	**630**	**646**	**648**	**652**					
R. flavipes	Y	R	R	Y	C	M	C	R	M	C	G	H	T	C	Y	K	S	S	Y	T	M	R	A	M					
R. hageni	C	A	A	S	C	A	Y	A	C	T	G	A	Y	T	T	T	G	C	T	C	A	G	A	C					
R. malletei	C	C	A	T	C	A	C	A	C	T	G	A	T	T	T	T	A	C	T	T	A	G	A	C					
R. nelsonae	C	A	R	Y	C	A	C	A	Y	Y	G	A	T	T	T	T	G	C	T	T	A	G	A	C					
R. virginicus	C	A	A	C	Y	A	C	A	C	T	R	A	T	T	T	T	R	C	Y	T	A	G	R	Y					
Cut site		✓												✓						×									

Table S4. Original interpretive framework for identifying eastern United States *Reticulitermes* termite species using PCR-RFLP applied to the mt*COII* gene, based on "training data" from NCBI's GenBank database. Expected fragment sizes produced by three restriction enzyme digests (performed one-at-a-time) for each of five recognized taxa are represented by check marks. Following application to a "test panel" of Southern Appalachian samples, the interpretive framework was refined to accommodate new polymorphisms (see Table 1 in the main text).

Restriction Enzyme	Fragment Sizes (bp)	Species				
		R. flavipes	***R. hageni***	***R. malletei***	***R. nelsonae***	***R. virginicus***
Rsa I	175, 201	✓	×	×	✓	✓
	48, 127, 201	×	×	✓	✓	×
	86, 115, 175	×	✓	×	×	×
Taq I	376	×	✓	✓	✓	✓
	153, 223	×	×	×	✓	×
	183, 193	×	×	×	×	×
	67, 126, 183	✓	×	×	×	×
	30, 67, 126, 153	✓	×	×	×	×
Msp I	376	✓	×	×	×	✓
	37, 339 *	✓	×	✓	×	×
	77, 299	×	✓	×	✓	×
	37, 40, 299	×	×	✓	×	×

* For *R. flavipes*, fragment sizes may instead be 38-bp and 338-bp, but since the 1-bp differences compared to those reported in the table are indistinguishable, only the shorter fragments are listed.

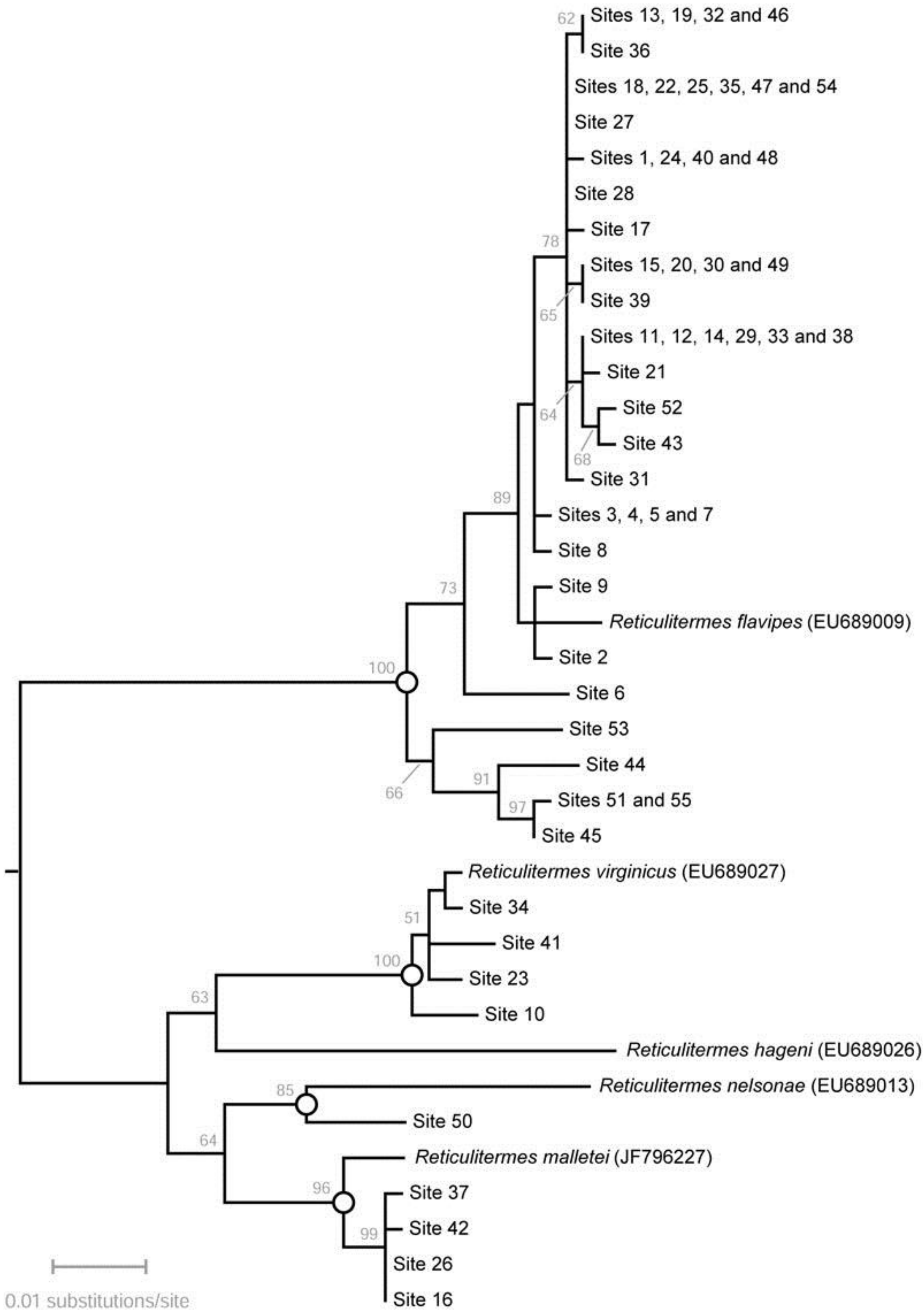

Figure S1. Phylogenetic relationships among *Reticulitermes* mt*COII* sequences from 55 new and five reference specimens (n = 37 non-redundant haplotypes, 607-bp each), estimated using Maximum Likelihood and the HKY+I model of nucleotide substitution. Shown here is the tree with the highest log likelihood. Numbers above nodes are bootstrap support values. Open circles on nodes mark the most inclusive well-support clades that contain only one reference taxon (the same groups were recovered using Maximum Parsimony; see Figure 2 of the main text).

References

1. Perdereau, E.; Bagnères, A.-G.; Bankhead-Dronnet, S.; Dupont, S.; Zimmermann, M.; Vargo, E.L.; Dedeine, F. Global genetic analysis reveals the putative native source of the invasive termite, *Reticulitermes flavipes*, in France. *Mol. Ecol.* **2013**, *22*, 1105–1119.
2. Austin, J.W.; Szalanski, A.L.; Uva, P.; Bagneres, A.G.; Kence, A. A comparative genetic analysis of the subterranean termite genus *Reticulitermes* (Isoptera: Rhinotermitidae). *Ann. Entomol. Soc. Am.* **2002**, *95*, 753–760.
3. Su, N.-Y.; Ye, W.; Ripa, R.; Scheffrahn, R.H.; Giblin-Davis, R.M. Identification of Chilean *Reticulitermes* (Isoptera: Rhinotermitidae) inferred from three mitochondrial gene DNA sequences and soldier morphology. *Ann. Entomol. Soc. Am.* **2006**, *99*, 352–363
4. Jenkins, T.M.; Basten, C.J.; Kresovich, S.; Forschler, B.T. Mitochondrial DNA sequence questions *Reticulitermes* sp. social structure (Isoptera: Rhinotermitidae). *Sociobiology* **1999**, *34*, 161–172.
5. Jenkins, T.M.; Dean, R.E.; Verkerk, R.; Forschler, B.T. Phylogenetic analyses of two mitochondrial genes and one nuclear intron region illuminate European subterranean termite (Isoptera: Rhinotermitidae) gene flow, taxonomy, and introduction dynamics. *Mol. Phylogenet. Evol.* **2001**, *20*, 286–293.
6. Cameron, S.L.; Whiting, M.F. Mitochondrial genomic comparisons of the subterranean termites from the genus *Reticulitermes* (Insecta: Isoptera: Rhinotermitidae). *Genome* **2007**, *50*, 188–202.
7. Ye, W.; Lee, C.Y.; Scheffrahn, R.H.; Aleong, J.M.; Su, N.Y.; Bennett, G.W.; Scharf, M.E. Phylogenetic relationships of nearctic *Reticulitermes* species (Isoptera: Rhinotermitidae) with particular reference to *Reticulitermes arenincola* Goellner. *Mol. Phylogenet. Evol.* **2004**, *30*, 815–822.
8. Yashiro, T.; Matsuura, K. Distribution and phylogenetic analysis of termite egg-mimicking fungi "termite balls" in *Reticulitermes* termites. *Ann. Entomol. Soc. Am.* **2007**, *100*, 532–538.
9. Lim, S.Y.; Forschler, B.T. *Reticulitermes nelsonae*, a new species of subterranean termite (Rhinotermitidae) from the southeastern United States. *Insects* **2012**, *3*, 62–90.
10. Sillam-Dussès, D.; Forschler, B.T. A dominant and undescribed species of *Reticulitermes* in Sapelo Island (Georgia, USA). *Sociobiology* **2010**, *56*, 137–147.

© 2015 by the authors; licensee MDPI, Basel, Switzerland. This article is an open access article distributed under the terms and conditions of the Creative Commons Attribution license (http://creativecommons.org/licenses/by/4.0/).
